# Online art therapy in elementary schools during COVID-19: results from a randomized cluster pilot and feasibility study and impact on mental health

**DOI:** 10.1186/s13034-021-00367-5

**Published:** 2021-03-06

**Authors:** Catherine Malboeuf-Hurtubise, Terra Léger-Goodes, Geneviève A. Mageau, Geneviève Taylor, Catherine M. Herba, Nicholas Chadi, David Lefrançois

**Affiliations:** 1 Department of Psychology, Bishop’s University, 2600 College St., Sherbrooke, QC J1M 1Z7 Canada; 2 Faculty of Medicine and Health Sciences, Université de Sherbrooke, Sherbrooke, Canada; 3 Department of Psychology, Université de Montréal, Montreal, Canada; 4 Department of Education and Pedagogy, Université du Québec à Montréal, Montreal, Canada; 5 Department of Psychology, Université du Québec à Montréal, Montreal, Canada; 6 Department of Paediatrics, Sainte-Justine University Hospital Centre, Montreal, Canada; 7 Department of Educational Sciences, Université du Québec en Outaouais, Gatineau, Canada

## Abstract

**Background:**

Emerging literature on the current COVID-19 crisis suggests that children may experience increased anxiety and depression as a result of the pandemic. To prevent such school and mental health-related problems, there is a timely need to develop preventive strategies and interventions to address potential negative impacts of COVID-19 on children’s mental health, especially in school settings. Results from previous child clinical research indicate that art-based therapies, including mindfulness-based art therapy, have shown promise to increase children’s well-being and reduce psychological distress.

**Objective:**

The goal of the present pilot and feasibility study was to compare the impact of an emotion-based directed drawing intervention and a mandala drawing intervention, on mental health in elementary school children (*N* = 22), in the context of the COVID-19 pandemic. Both interventions were group-based and delivered online and remotely. A pilot study using a randomized cluster design was implemented to evaluate and compare both interventions in relation to child anxiety, depression, inattention and hyperactivity symptoms.

**Results:**

Analyses of covariance revealed a significant effect of the type of drawing intervention on levels of inattention, after controlling for baseline levels. Participants in the emotion-based directed drawing group showed lower inattention scores at post-test, when compared to participants in the mandala group. Post-hoc sensitivity analyses showed significant decreases in pre-to-post scores for levels of hyperactivity for the complete sample.

**Conclusion:**

Overall, results from this pilot and feasibility study showed that both an emotion-based directed drawing intervention and a mandala drawing intervention may be beneficial to improve mental health in elementary school children, in the context of the current COVID-19 pandemic. From a feasibility standpoint, results indicate that the implementation of both interventions online and remotely, through a videoconference platform, is feasible and adequate in school-based settings. Further work incorporating larger sample sizes, longitudinal data and ensuring sufficient statistical power is warranted to evaluate the long-term impact of both interventions on children’s mental health.

## Background

Emerging literature on the current COVID-19 crisis suggests that children may experience increased anxiety and depression in the context of the COVID-19 pandemic [23]. Specifically, emotional and behavioral problems arising in children who have been or are still confined to their homes have been reported [14, 17, 21, 24, 29]. A lack of social interactions, boredom and family tensions may all contribute to the emergence of these problems [21]. Early evidence indicates that the effects of the COVID-19 pandemic are particularly salient among children who have a pre-existing psychological disorder [8].

Recent data suggests that elementary school children have experienced an increase in school and psychosocial adaptation problems, in the context of the current pandemic [23]. It has been suggested that these problems may impede academic achievement and school perseverance [29] which may have long-lasting impacts extending well beyond the current pandemic. To prevent such school and mental health-related problems, there is a timely need to develop preventive strategies and interventions to address potential negative impacts of COVID-19 on children’s mental health, especially in school settings.

Results from previous child clinical research indicate that interventions based on social-emotional learning could potentially increase children’s well-being and reduce psychological distress, while encouraging perseverance and academic achievement in school [17]. Art-based therapies, including mindfulness-based art therapy, have shown promise in this regard, although to date they have been mostly delivered in-person and not online [6, 12].

In the current context of the COVID-19 pandemic, online psychological interventions delivered at school (either in-class, or as a virtual learning activity) have the potential of leading to wide-scale provision of psychological support to high numbers of children. Available empirical evidence from previous natural disasters and pandemics indicate that providing children with online psychological services, such as tele-health, have significant positive effects on their mental health [13]. Preliminary results have suggested that online-based group interventions can be beneficial to improve mental health of elementary school students, in the context of the current COVID-19 pandemic ([22], submitted). The goal of this study was thus to compare the impact of two online, group-based, art- and mindfulness-based drawing interventions on elementary school students’ mental health, within the context of the current COVID-19 pandemic.

### Art-based interventions for youth

Although art-based interventions and art therapy initially drew their theoretical framework in psychoanalytic perspectives, recent work has shown influence by a variety of theoretical paradigms, such as cognitive-behavioral therapy and social-emotional learning [5, 28]. The use of art in in-person clinical contexts with youth has been shown to facilitate and encourage self-expression, discussion and awareness of emotions, through an alternative means of communication [28]. This, in turn, encourages verbal expression of emotions and allows for easier communication of difficult emotions [9].

Although there is a paucity of empirical research on the impacts of in-person art therapy for children, preliminary evidence of its usefulness in children who are chronically ill suggests it holds promise in improving mental health, namely by decreasing anxiety and improving overall quality of life [2, 11]. These improvements are explained in part by the fact that art helps children gain a sense of control over their decisions, while providing an outlet to share emotions, in a context where there usually is little or none [11]. Parallels can be drawn between the lack of control over one’s life in the context of a chronic illness and in the current context of the COVID-19 pandemic. As such, it appears likely that art-based interventions could have similar benefits on children’s mental health during this global crisis, although the available empirical evidence only applies to in-person interventions.

Previous research on the impacts of in-person art-based interventions conducted in school settings indicate that they promote awareness and self-understanding in children with learning disabilities, which in turn fosters better emotional and social adjustment [12]. Furthermore, in a group setting, art therapy was associated to decreases in hyperactivity and internalizing behaviors, while improving social skills, in a sample of children with autism [10]. However, although preliminary evidence suggests that in-person art-based interventions are beneficial in clinical populations, further research is warranted to determine if similar effects can be found using an online modality, in non-clinical populations and as a preventive measure to reduce psychological distress, namely with elementary school students.

### Mindfulness-based art therapy

Mindfulness can be defined as paying attention open-mindedly to the present moment, without judgement [18]. Mindfulness-based interventions tailored to children have shown benefits for reducing psychological distress and improving overall mental health when implemented in elementary school classrooms, specifically with regards to anxiety, depression and inattention [4, 30]. Although youth mindfulness studies report results from interventions which use formal meditative practices, recent research has started to explore alternative, informal mindfulness practices and interventions, including online deliveries, that may be beneficial for youth mental health, for example, through artistic expression [5–7].

In order for a practice to be considered mindful, it must include a structured component that encourages the focus of attention [15]. As such, mindful art making is defined as a practice in which both the physical and creative processes of art are included, along with a specific intent to concentrate on the unfolding of the present moment [3]. Drawing-based interventions, such as mandala drawing, are considered a form of mindfulness-based art therapy, and are easily implemented in school settings. Past research with adults and teenagers has shown in-person mandala drawing interventions to be beneficial for decreasing anxiety in school settings and clinical contexts [19, 27]. However, a small study conducted with elementary school children suggested that in-person mandala drawing was no more effective than free drawing (non-mindful) in reducing test anxiety [3]. Thus, further research is warranted to establish if mandala drawing represents a viable intervention to improve youth mental health, specifically in school settings.

## Present study

The goal of the present pilot and feasibility study was to compare the efficacy of an emotion-based directed drawing intervention and a mandala drawing intervention, both group-based, delivered online and remotely, for mental health in elementary school children, in the context of the COVID-19 pandemic. Specifically, the impact of both interventions on anxiety, depression, inattention and hyperactivity symptoms was compared. To do so, a randomized cluster pilot study was implemented. Based on the existing literature and the fact that the emotion-based intervention was directed, whereas the mandala intervention was not and more attention-focused, we hypothesized that the children in the emotion-based intervention group would have lower anxiety, depression, inattention and hyperactivity than the ones in the mandala intervention. Furthermore, our hypothesis was based on the fact that in the emotion-based drawing intervention, participants were more explicitly talking about feelings, which we expected to help them better process this information.

## Methods

All available students from two classrooms of 4th to 5th grade (*N* = 22; M_age_ = 11.3 years old; equal number of boys and girls) from an elementary school in the Eastern Townships region in Quebec, Canada, took part in this study and were randomly allocated to either the mandala drawing group (1 classroom of 5th grade students; *N* = 8) or the emotion-based directed drawing group (1 multi-age classroom of 4th and 5th grade students; *N* = 14; please refer to Table 1). Interventions occurred simultaneously in the months of May and June of 2020, during the COVID-19 pandemic and after the gradual reopening of the school, following a 6 weeks confinement period. In the province of Quebec where this study took place, government authorities issued a province-wide lockdown, including all non-essential store closings, elementary and high school closings, starting on March 13th. These restrictions were in place until the beginning of the month of May, for schools outside of the greater Montreal area (where schools remained closed until the end of the 2019–2020 school year because of an increased infection rate). There was no active remote teaching for the first few weeks of this confinement. Remote teaching (and learning) took place during the month of April, where elementary school teachers were asked to contact their students individually first, and then to organize online classes through videoconferencing platforms. As such, although the specific details of teaching delivery varied from one region of the province to the next, overall, all elementary school students received some form of remote teaching during the month of April. Online learning for elementary school students did not include long interactions online, but rather could have differed depending on the school, and tended to be once per day for a 45-min period, given the age of the children. Upon school reopening in May in the Eastern Townships where this study took place, Quebec’s Public Health officials imposed strict guidelines: elementary schools were only allowed to reintegrate 50% of their students, in order to respect social distancing instructions. The province of Quebec presented with the highest infection rate in Canada, with a significant amount of outbreaks and casualties in long-term health care facilities and retirement homes.

**Table 1:** Descriptive statistics and sample distribution

Classroom grade	N	Mean age	Condition
Multi-age 4th–5th	14	11.2	Emotion-based
5th	8	11.5	Mandala
Total sample	22	11.3	
Girls	11		
Boys	11		

Informed consent was obtained from parents of participants and teachers, and assent was obtained from participating children. Randomization occurred immediately after completion of pre-intervention measures. Students completed pre-intervention (1 week before the beginning of the intervention) and post-intervention (1 week after the end of the intervention) measures. Two research assistants assisted students remotely in completing the questionnaire, namely by reading all question items out loud to the group and answering students’ questions. There was no attrition in this study.

### Procedure

As part of this study, two distinct group-based interventions were implemented. Both interventions lasted 5 weeks during the months of May and June 2020 (1 session per week), with each session lasting approximately 45 min. Sessions occurred through a secure, password protected, video conferencing platform, during which research assistants would log on remotely and join students in their classroom, using the classrooms’ smart board. The classroom would stay on the videoconference call for the whole duration of the drawing activity. Teachers were present in the classroom with the students during the sessions. All students had physically returned to school at the time of this study. Sessions were led by two undergraduate psychology students with previous experience leading mindfulness-based interventions in elementary schools as well as prior research experience related to art-based therapy. Research assistants worked as a pair and led all groups together for both modalities. They were blinded to the research hypotheses. Structured clinical supervision was offered to research assistants online and weekly by a licensed child clinician (CMH, first author of this study) throughout this project, during which research assistants were provided a safe space to talk about and process the children’s reactions to the proposed activities, as well as suggested ways of responding to some of the children’s comments to ensure empathic and active listening, as well as validation of their emotions. Furthermore, supervision sessions were also used to monitor both interventions’ implementation fidelity.

The emotion-based directed drawing intervention that was used in this project consisted of five weekly sessions during which children were instructed to complete varying drawing activities, all targeted towards exploring emotions and/or to provide an occasion to discuss COVID-19. Details of the weekly sessions can be found in Table 2. This intervention was based on Elise Gravel’s (a children’s book author and illustrator) *How Do You Doodle? Drawing my Feelings and Emotions* and on the author’s recently published COVID-19 themed drawing worksheets for children, available on the author’s website (http://elisegravel.com/livres/dessins-a-colorier/). Specifically, students were asked to make drawings related to fear, worry, irritation, but also to draw how they were feeling. Pandemic-specific activities included drawing viruses and a cure to COVID-19. Group discussions followed each activity, during which children were invited to share their thoughts, emotions and overall reactions to their drawings. The emotion-based drawing intervention used in this study was not considered as a subtype of mindfulness-based art therapy, namely because it lacked a mindfulness component. Indeed, the emotion-based drawing intervention did not incorporate any specific instruction to paying attention in the present moment, nor any formal practice of mindfulness meditation (which was not included either in the mandala intervention described below). However, it did provide a good forum for children to think about their feelings and emotions.

**Table 2:** Content of emotion-based drawing intervention

Session	Content
1	● COVID-19/story of a virus Comic strip ● Looking inside yourself—draw how you feel
2	● Recipe for a nice day ● Drawing viruses with funny names
3	● Fear: draw what you are afraid of in a bottle and put a cork in it ● Irritation: draw what aggravates you and throw it in the garbage can
4	● Worry: draw makes you anxious and where you feel it in your body
5	● Weekly forecast: what’s the forecast in your heart today? ● Draw your COVID-19 cure

The mandala drawing intervention consisted of 5 different mandala drawing sessions taken from the *CBT Art Activity Book* [16]. Students were simply asked to draw one mandala per session, without further instructions. No COVID-19 specific activities were included in this intervention. Following the drawing of their mandalas, children had the opportunity to discuss and share their reflections related to their drawings as a group.

### Measures

Measures were administered 1 week prior the beginning of both interventions and 1 week after the end of both interventions. Both interventions started at the same time. Mental health symptoms were assessed using the *Behavior Assessment Scale for Children-3rd edition* (BASC III) [25]. To minimize testing time, participants completed selected items from the anxiety (3 items, e.g., “I worry about little things”; original subscale comprised of 11 items), depression (5 items, e.g., “I feel sad”; original subscale comprised of 10 items), inattention (3 items, e.g., “I forget to do things”; original subscale comprised of 9 items) and hyperactivity (2 items, e.g., “I have trouble sitting still”; original subscale comprised of 8 items) subscales. Items were selected following discussions with the research team members. Internal consistency was variable, ranging from poor to good, across subscales (α anxiety_pre/post_ = 0.77/0.79; α depression_pre/post_ = 0.65/0.76; α inattention_pre/post_ = 0.93/0.66; α hyperactivity_pre/post_ = 0.74/0.72) in this sample. Individual scale scores were used in the analyses.

Participants also completed seven items from the Mindful Attention Awareness Scale for Children [20] (7 items; e.g., “I find it difficult to stay focused on what is happening in the present moment.”). In this scale, all items are reverse scored. Internal consistency was good (α_pre/post_ = 0.83/0.85) in this sample.

### Data analysis

We tested our hypotheses using analyses of covariance (ANCOVAs), which can maximize statistical power in randomized controlled pilot studies, and which allowed for a comparison of post-intervention scores between each group, using pre-intervention scores as covariates [26]. Effect sizes, Partial η^2^ scores, were computed in order to assess the magnitude of the observed effects. Post-hoc sensitivity analyses using paired t-tests were also completed to examine changes from pre-to-post intervention within the full sample.

## Results

**Fig. 1:**
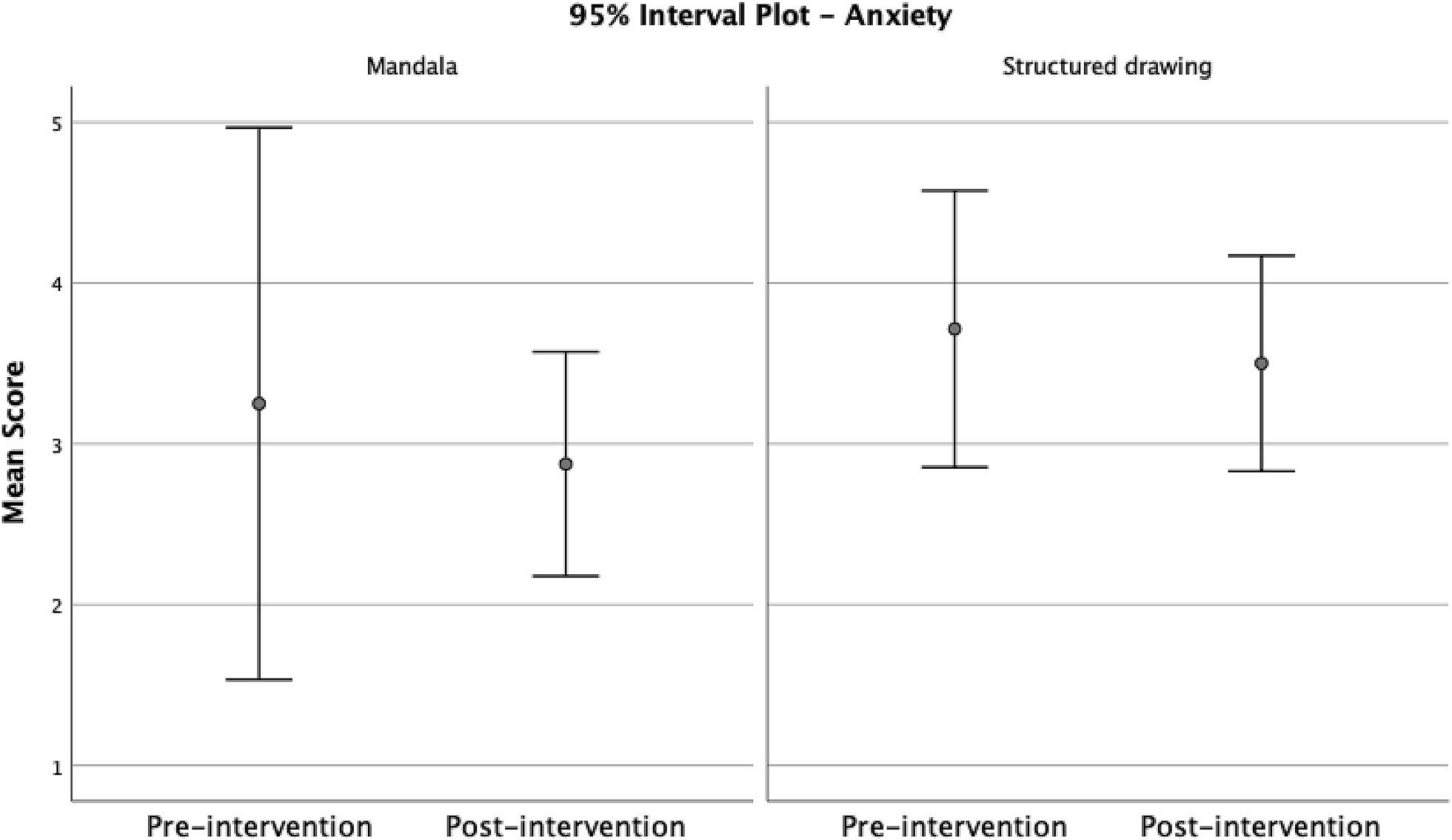
Pre-to-post mean scores and 95% confidence interval across groups and variables

**Fig. 2:**
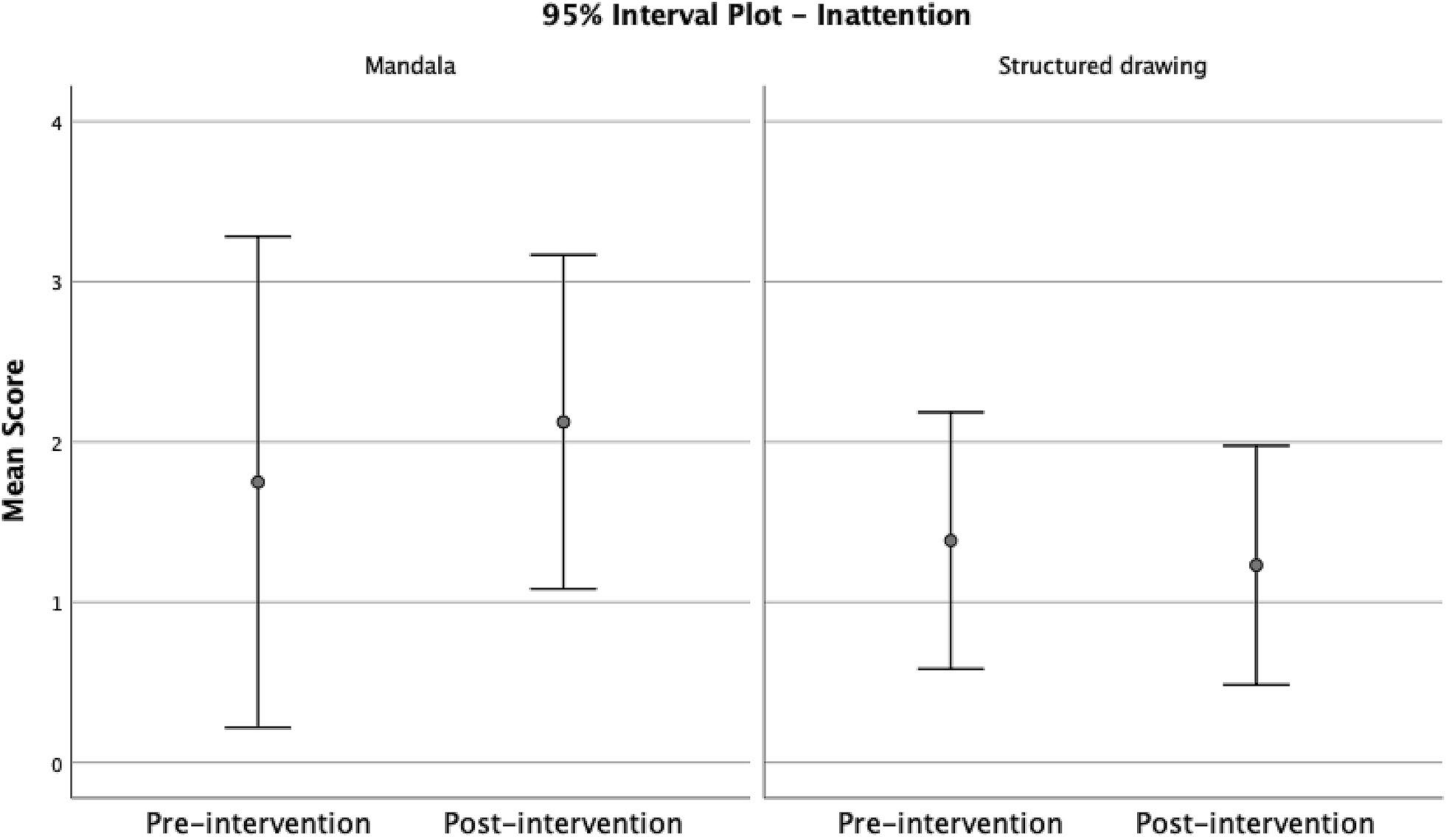
Pre-to-post mean scores and 95% confidence interval across groups and variables for inattention

**Fig. 3:**
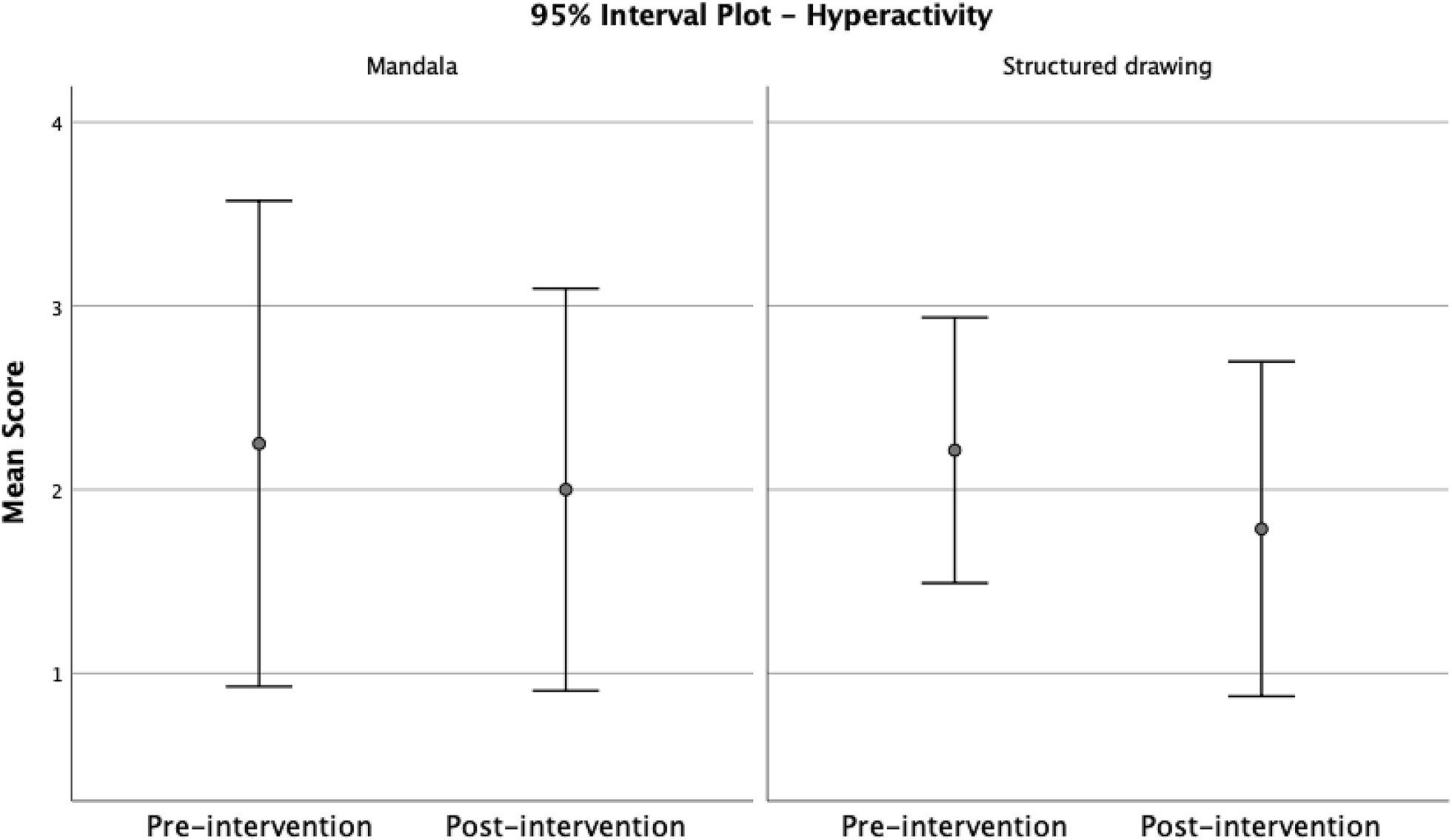
Pre-to-post mean scores and 95% confidence interval across groups and variables for hyperactivity

**Fig. 4:**
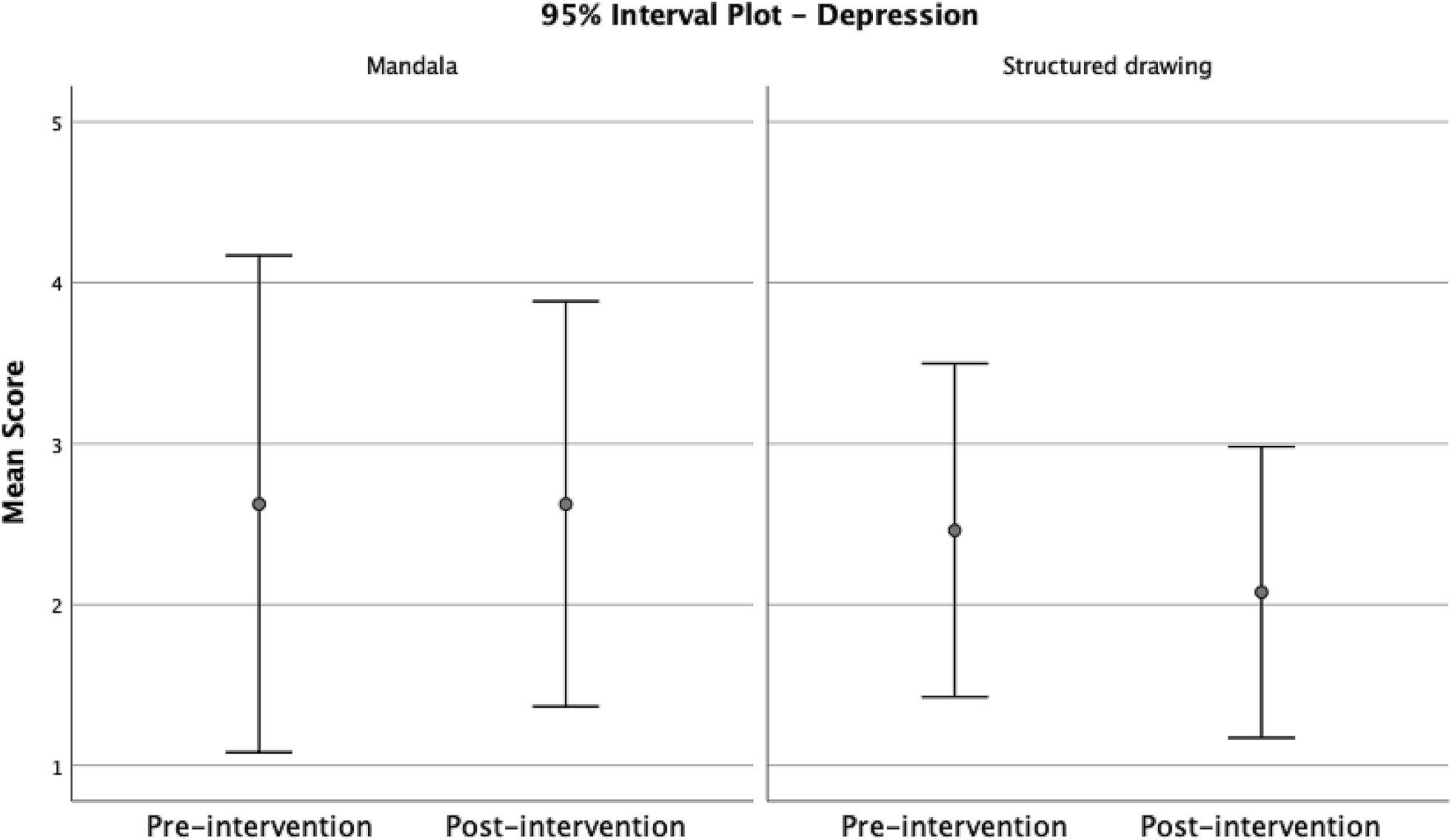
Pre-to-post mean scores and 95% confidence interval across groups and variables for depression

**Fig. 5:**
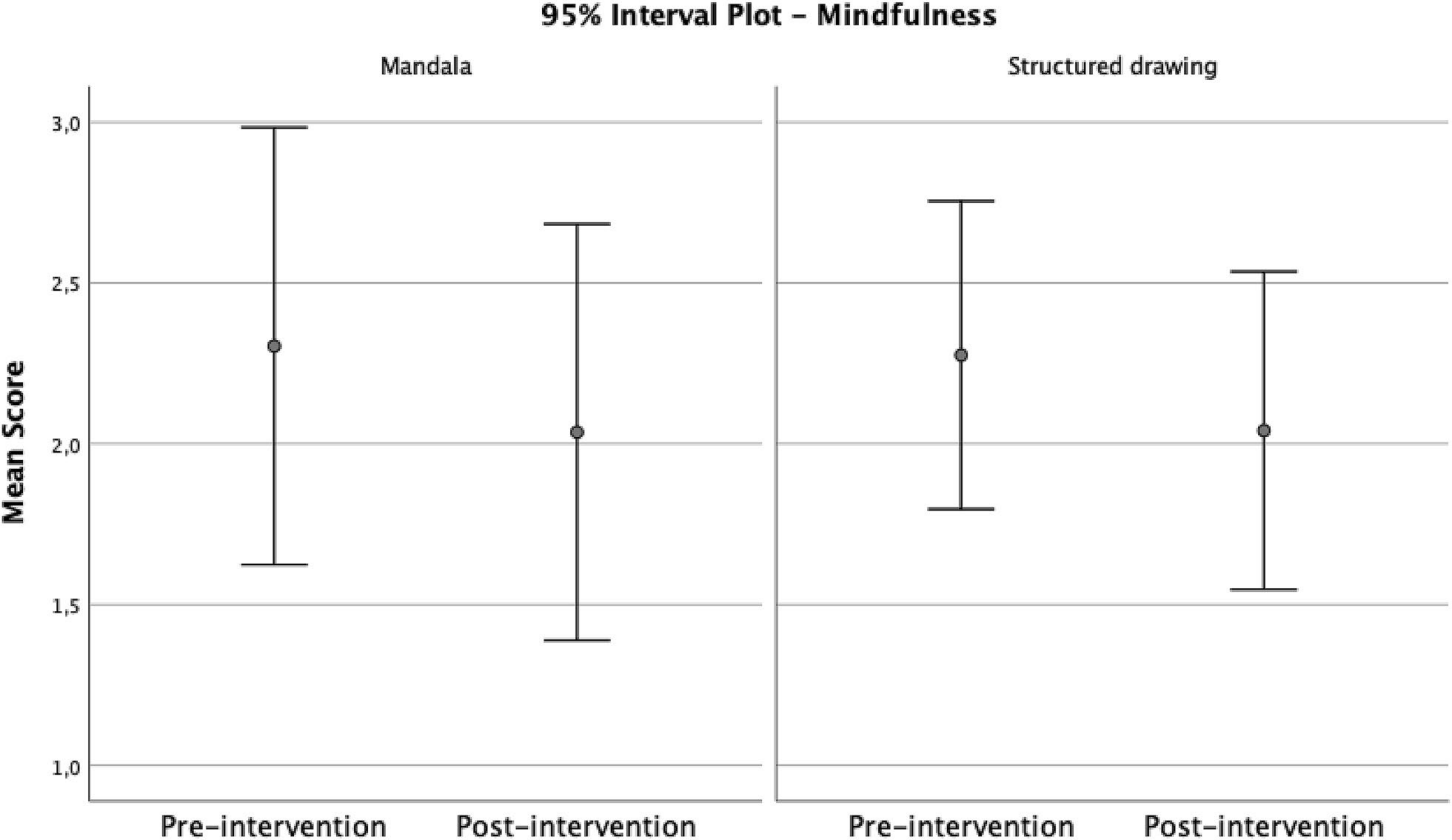
Pre-to-post mean scores and 95% confidence interval across groups and variables for mindfulness

Given the unequal size of both groups, we tested for homogeneity of variance for all analyses. Results from Levene’s tests of equality of error variances showed that our data met the homogeneity of variance assumption. ANCOVAs revealed a significant effect of the type of drawing intervention on inattention symptoms (F (1, 20) = 4.15, *p* = 0.05, partial *η*^2^ = 0.19), after controlling for baseline levels of symptoms. Participants in the emotion-based directed drawing group showed lower inattention scores at post-test (M_post, adjusted for baseline_ = 1.32), when compared to participants in the mandala group (M_post, adjusted for baseline_ = 1.97). However, sensitivity analyses using paired t-tests did not show significant pre-to-post changes in inattention scores in participants from each group (*p*_emotion-based_ = 0.43; *p*_mandala_ = 0.35). We found no impact of type intervention group on levels of anxiety, depression, hyperactivity or mindfulness (please refer to Tables 3, 4 and Figs. 1, 2, 3, 4, 5).

**Table 3:** Means and standard deviations for anxiety, depression, inattention, hyperactivity and mindfulness

Dependent variable	Mandala group		Emotion-based drawing group	
	Pre-test (SD)	Post-test (SD)	Pre-test (SD)	Post-test (SD)
Anxiety	3.25 (2.05)	2.87 (.83)	3.71 (1.48)	3.5 (1.70)
Depression	2.62 (1.84)	2.62 (1.50)	2.46 (1.71)	2.07 (1.49)
Inattention	1.75 (1.83)	2.12 (1.24)	1.38 (1.32)	1.23 (1.23)
Hyperactivity	1.37 (1.4)	2.00 (1.3)	2.21 (1.25)	1.78 (1.57)
Mindfulness	2.30 (0.81)	2.03 (0.77)	2.27 (0.83)	2.04 (0.85)

**Table 4:** Results of ANCOVA for anxiety, depression, inattention, hyperactivity and mindfulness

Variable	df	F	*P*	Partial η^2^
Anxiety	1	1.33	0.26	0.07
Depression	1	1.07	0.68	0.04
Inattention	1	4.15	**0.05**	**0.19**
Hyperactivity	1	0.22	0.64	0.01
Mindfulness	1	0.006	0.94	0.00

Bold values indicate statistically significant p values (p ≤ 0.05)

Post-hoc sensitivity analyses showed significant decreases in pre-to-post scores for levels of hyperactivity (*t*(21) = 2.01, *p* = 0.05) for the complete sample. It thus seems that participants from both groups showed a decrease in scores from pre-intervention (M_pre total sample_ = 2.22) to post-intervention (M_post total sample_ = 1.86).

## Discussion

The goal of the present pilot and feasibility study was to compare the impact of two group-based drawing interventions, implemented remotely and online, on elementary school children’s mental health, in the context of the COVID-19 pandemic. Although results from our primary analyses seem to indicate that the emotion-based directed drawing intervention may be more salient for inattention symptoms than the mandala intervention, sensitivity analyses did not indicate significant pre-to-post variations in inattention scores among participants from the emotion-based drawing intervention group. This leads us to question the robustness of these results and thus warrants caution in their generalization. Furthermore, post-hoc sensitivity analyses also showed significant decreases from pre-to-post intervention in hyperactivity scores in our total sample. As such, results from this study indicate that both interventions were associated with mental health gains, namely with regards to inattention, in this sample. Hence, both a directed, emotion-based intervention and a mandala intervention appear valuable options with elementary school students. Findings need to be interpreted very cautiously as there was little difference between groups in this study.

Nonetheless, results from this preliminary study may suggest that a directed, emotion-based drawing intervention could be useful to alleviate symptoms that may be attributed to increased levels of psychological distress, such as inattention, in elementary school children, in the current context of the COVID-19 pandemic. Although results from this study are underpowered due to the study’s small sample size, the decreases found in inattention scores are supported by a moderate to large effect size. Indeed, giving children the opportunity to reflect on their feelings, as well as providing them with a safe space in which to process what they understand of the COVID-19 pandemic and how they feel about it, through artistic expression, may be helpful to improve attention capacities. Nonetheless, it is also important to note that students in the mandala condition also showed improvements on mental health measures, such as hyperactivity, which indicates that a non-directed approach may also be useful to alleviate psychological distress that is associated to COVID-19.

It should be noted that across both groups, discussions had approximately the same duration. As such, given the different nature of both interventions and the fact that the mandala intervention was not intended to discuss COVID-related events, it is possible that post-drawing discussions may be driving the observed differences in terms of inattention symptoms in the emotion-directed group. This being said, given that children in both groups knew each other well, perhaps overall improvements in both groups reflects the cohesion of discussion and group expression. This may explain why students in the mandala group showed similar improvements to those in the emotion-based condition, namely with regards to hyperactivity symptoms. Indeed, the ability to express feelings is easier to facilitate in groups in which students know each other well, which was the case in this study. This being said, given the fact that we did not have a third control group receiving no intervention in this study, then it is difficult to say whether improvements on hyperactivity in the full sample can be explained by regression towards the mean, or whether both groups improved because students became more used to the COVID situation, in comparison to 5 weeks earlier (at the beginning of the study). This could be especially true given the rapid evolution of events in this pandemic context. Finally, improvements in the overall sample could potentially have been due to the situation ‘normalising’ a bit more for both groups with returning to school.

Nonetheless, given the fact that school teachers, professionals and researchers in child mental health alike will most likely continue to face significant issues with regards to student mental health in the upcoming year, these preliminary findings indicate that both drawing-based interventions, which can be implemented easily and with minimal training involved, may be interesting options to provide emotional support to children in school settings. These results also lend support to previous studies in which art-based interventions, including mindfulness-based art therapy, had positive effects on children’s mental health [9, 10, 12].

In children of elementary school age, manifestations of inattention, such as being easily distracted, forgetful or having difficulty paying attention to the teacher, can often be the primary manifestation of underlying anxiety and depression [1]. This may lead to mislabeling or misdiagnosis of either condition as attention deficit hyperactivity disorder (ADHD). Thus, it is possible, although results did not indicate a significant impact of either intervention on anxiety and depressive symptoms, that pre-to-post changes in inattention scores in participants from the emotion-based drawing group may reflect, at least in part, some improvements on these variables. It is also possible that children themselves were less attuned to their own variation in depression and anxiety symptoms, which may explain the lack of significant results in this regard. It is also possible that, as the children returned to school and used to its inherent structure, that their attention capacities improved. Using a larger number of scales and items for each variable would could help in solving this issue in future work. Indeed, the small number of items that were administered most likely reduced the sensitivity of our measures, which may also have impacted our internal validity. Perhaps new significant pre- to-post changes would be detected with a larger number of administered items. Including teacher and parent reported data would also help in this regard, as children, especially younger ones, may not always have the best insight to report on their own variations of symptoms. Finally, further work, with larger sample sizes, could help confirm or infirm this hypothesis.

Post-hoc sensitivity analyses conducted on pre-to-post changes in our total sample showed significant decreases in hyperactivity scores. These results lend further support in showing the promising impact of both drawing interventions on children’s mental health. It is quite possible that the lack of statistical power and unequal groups prevented us from seeing significant between-group differences on hyperactivity symptoms, although this remains to be further investigated.

From a feasibility standpoint, both interventions were implemented with very few technical difficulties. Indeed, while we did not conduct formal qualitative interviews of measures of acceptability and feasibility, informal discussions (anecdotal information) with teachers indicated they were highly motivated in ensuring that this online research project could run smoothly and were keen to assist the research team, for instance, by facilitating group discussions and questionnaire completion. Feedback collected from teachers and students alike shows that both interventions were well accepted and appreciated, especially by teachers, who had very clearly expressed a need to address the COVID-19 pandemic and its consequent psychological impacts on their students upon reopening of their school. Teachers also mentioned that we were able to provide a playful and creative approach to discussing current events and alleviating psychological distress, through a stimulating art-based intervention, especially for students in the emotion-based drawing group. Future work could aim to evaluate acceptability and feasibility in a more formal manner to document this aspect of the research. Given that our research team does not anticipate that research assistants will be allowed in schools during the 2020–2021 school year, knowing that the online modality is feasible and yields encouraging results on youth mental health is quite encouraging.

Results from this study do not enable us to conclude whether both interventions are effective or not in alleviating anxiety or depressive symptoms and whether one intervention is more effective than the other in this regard. It is also possible that the 5-week duration of both interventions was too short to provide significant impacts on mental health and significant group differences. Indeed, it is recommended that future work aiming to replicate this study may test longer interventions’ impact on youth mental health. Nonetheless, results offer preliminary evidence that both an emotion-based directed drawing intervention and a mandala drawing intervention can be helpful to decrease manifestations of psychological distress in elementary school children. Although the lack of statistical power is one of our study’s main limitations, our research team felt it was relevant to publish these preliminary, yet encouraging results, in light of the time-sensitivity of the COVID-19 pandemic. Given the ease of a remote and online implementation of these interventions, disseminating these results could stimulate future studies for those interested in exploring avenues to improve child mental health, especially in the context of the COVID-19 pandemic.

Logical next steps for our study include recruiting a larger sample size and including longitudinal data which could help further the knowledge on the usefulness of mandala and emotion-based drawing interventions to address psychological distress in youth, as well as their long-term impact. Variations in pre-to-post mean anxiety and depression scores, albeit not statistically significant, seem to indicate that the emotion-based directed drawing intervention may be useful to improve such symptoms, although this remains to be confirmed in future, more robust work. Finally, the comparative and long-term impact of both interventions also remains to be investigated in more detail.

## Conclusion

Overall, results from this pilot and feasibility study showed that both an emotion-based directed drawing intervention and a mandala drawing intervention may be beneficial to improve mental health, namely inattention and hyperactivity, in elementary school children, in the context of the current COVID-19 pandemic. From a feasibility standpoint, results indicate that the implementation of both interventions online and remotely, through a videoconference platform, is feasible and adequate in school-based settings. Further work incorporating larger sample sizes, longitudinal data and ensuring sufficient statistical power is warranted to evaluate the long-term impact of both interventions on children’s mental health.

## Data Availability

All datasets used and analyzed in the current study are available on request from the study’s first author.
